# Dr. Talcott’s Paper

**Published:** 1897-11

**Authors:** B. Fincke


### DR. TALCOTT'S PAPER.

B. FINCKE, M. D.

The paper is a sign of progress because it brings a topic to discussion which formerly was silenced or only sneered at.

It is nothing but the acknowledgment of the Hahnemannian dynamic theory, which was propounded before the natural philosophers proclaimed the mechanical theory, based upon the principles of conservation of forces, correlation of forces, and persistence of force.

Every medicinal substance or drug contains the medicinal potential energy, which, on being acted upon by inert vehicles, changes into dynamic energy, and is transferred upon them imparting to them its nature. If imparted to the living body in health and disease it changes likewise into dynamical energy, and produces and heals symptoms distinctly observable in the direction which the quality of the medicinal force indicates.

Functional cases can also depend upon pathological lesions, and therefore the division is not justifiable. But physiological action is nothing but the dynamic action upon the life-force which directs the dynamic energy to the parts according to homœopathic law.

The impression of the homœopathic prescription, therefore, is not psychological but physiological mediated by the life-force.

It does not follow that the only healing medicinal action is its direct or primary action, because it depends upon the sensibility and susceptibility of the person acted upon on one side, and on the potency on the other side whether symptoms produced by it will occur primarily or secondarily, or at any time of its duration.

It does not follow also that the more dilute dose (better the higher potency) insures a more stable cure. For it depends upon the susceptibility of the patient together with the exact simility of the remedy in selection of symptom and potency whether the equalization of the medicinal and pathogenetic forces will be accomplished, from which it follows that every case as it requires the proper selection according to symptoms—simility also requires the proper selection of the potency according to the sensitivity and susceptibility of the patient.

Thus the medicinal force of the crude substance may as well be the proper potency as a millionth, and in this lies the greatest difficulty of healing. Since, however, experience teaches that the crude substances and low potencies as a general rule do not act as safely or permanently as the higher potencies, the latter are to be preferred as being in their action more similar to the life-force and unencumbered by the matter of the drug substance.

The purpose of the paper is not reached, because what is said before only confirms the Hahnemannian dynamic theory afterward, by the natural philosophers termed the mechanical theory. As this is sufficient, and explains the potentiation of medicinal forces and their action upon the living body in health and disease it is not necessary to plunge into fields of investigation which lead into uncertainties and speculations. This is avoided by the Hahnemannian dynamism, which, indeed, leads into a purer practice and creates confidence in our potencies conveyed by a thorough knowledge of their nature and action to the practical homœopathician.

EMMA D. WILCOX,

*Secretary.*

Adjourned.

